# Prevalence of Insomnia and Sleep Patterns among Liver Cirrhosis Patients

**DOI:** 10.5334/jcr.aa

**Published:** 2014-11-19

**Authors:** Hamdan AL-Jahdali, Abdullah Al Enezi, Ahmed E. Anwar, Abdullah AL-Harbi, Salim Baharoon, Abdulrahman Aljumah, Abdullah Shimemeri, Khaleid Abdullah

**Affiliations:** Department of Medicine, Pulmonary Division, and Sleep Disorders Center. King Saud bin Abdulaziz University for Health Sciences, Riyadh, Saudi Arabia; Department of Epidemiology and Biostatistics, College of Public Health and Health Informatics, King Saud bin Abdulaziz University for Health Sciences, Riyadh, Saudi Arabia; Departments of Hepatobiliary Surgery and Liver Transplantation. King Saud bin Abdulaziz University for Health Sciences, Riyadh, Saudi Arabia

**Keywords:** Liver cirrhosis, insomnia, sleep disturbances, hepatitis C, hepatitis B, Child-Pugh scores

## Abstract

**Background:**

Few studies are available regarding the prevalence of sleep disturbance in cirrhotic patients without overt hepatic encephalopathy. This study aimed to assess the prevalence of insomnia in stable liver cirrhosis patients who are attending the outpatient clinics at King Abdulaziz Medical City, Riyadh (KAMC-KFNGH).

**Methods:**

A cross-sectional study enrolled 200 stable patients with confirmed liver cirrhosis. We used the ICSD-2 definition to assess the prevalence of insomnia. We also collected information about sleep patterns, demographic data, the underlying cause of liver cirrhosis and the severity of liver cirrhosis using Child-Pugh scores (CTP).

**Results:**

The mean age was 58.9 (SD ± 12.2) years. Hepatitis C was the most common (60.2%) cause of liver cirrhosis among respondents. The prevalence of insomnia was 42% (84/200). Univarite analysis shows association between coffee intake and the presence of insomnia (56.9% vs. 35.9%, p-value = 0.006). The prevalence of insomnia was higher in hepatitis C (51.7%) compared to hepatitis B (36.8%) and other hepatitis (15%), p-value = 0.001. There was a significant relationship between severity of liver cirrhosis (CTP-A, CTP-C, CTP-B) and prevalence of insomnia: 55%, 36.1% and 32.1% respectively, p-value = 0.009. Insomniac patients were significantly older than non-insomniac (61.6 ± 12.0 vs. 57.0 ± 12.0 years, p = 0.008). Results from the multivariate stepwise analysis showed coffee intake (OR=2.7), hepatitis C (OR = 7.2), CTP-A (OR = 1.9), excessive daytime sleepiness (OR = 5.3) and short sleep duration (OR = 5.7) were the most strongly associated with the presence of insomnia.

**Conclusion:**

Our study showed a high prevalence of insomnia in patients with liver cirrhosis.

## Background

Sleep disturbances are common and were recognized as one of early features of cirrhotic patients with hepatic encephalopathy (1,2,3). Although early studies revealed that inverse sleep pattern is an early sign of hepatic encephalopathy, Montagnese and colleagues did not find any correlation between the circadian rhythm abnormalities and hepatic encephalopathy (4). Few studies are available regarding the prevalence of sleep disturbance in cirrhotic patients without overt hepatic encephalopathy (1,2,5,6). These studies on the prevalence of sleep disturbance revealed wide variations ranging from 27–70 per cent (1,2,4,5,6). The most common sleep disorders reported in patients with liver cirrhosis are poor sleep quality, frequent awakening, difficulty falling asleep after awakening, prolonged sleeping latency, delayed bedtime, delayed wake-up time, excessive daytime sleepiness and preference for evening activities (1,2,3,4,6,7,8,9,10,11,12,13,14). Unfortunately, sleep disorders among liver cirrhosis patients are not only under-diagnosed and poorly managed, but they are also associated with poor survival rates (5,8,15).

The exact mechanism of sleep disturbance in liver cirrhosis is still a controversial issue in the literature. Many hypotheses have been proposed to explain the origin of sleep disturbance in liver cirrhosis patients without encephalopathy but none of them has completely demonstrated a strong association. In liver cirrhosis patients, the diurnal plasma melatonin profile showed a significant delay in the onset of plasma melatonin release and in its nocturnal peak level (14,15,16,17,18). Furthermore, studies have shown improvement of melatonin and circadian rhythm post liver transplants (18). This disturbance of the melatonin profile may reflect the changes in the circadian rhythm. However, Rodrigue and colleagues reported sleep disturbance in approximately 55 per cent of pre-transplant liver cirrhosis patients and this disturbance did not differ significantly post liver transplant (19). Other studies suggested desynchronization of circadian rhythm due to decreased activities of the retino-hypothalamic system in conjunction with melatonin level and toxin effect on the brain due to metabolic disturbance responsible for sleep disturbance in liver cirrhosis patients (4,5,20).

Most of the reported studies about sleep disorders in liver cirrhosis-assessed patients are for the sleepwalking pattern, and there are no studies specifically done to address symptoms of insomnia in particular, using a very well-defined criteria. Furthermore there is limited information about the association of insomnia and the severity of liver cirrhosis or underlying etiologies of cirrhosis (4,21).

Insomnia is characterized by one or more of the following symptoms: difficulty falling asleep (‘sleep onset insomnia’), difficulty staying asleep (‘sleep maintenance insomnia’) and early awakening or poor sleep quality (‘nonrestorative sleep’) (22). Insomnia is primarily a clinical diagnosis and is most frequently diagnosed using data obtained from patient histories and sleep diaries. The ICSD-2 defines insomnia as a difficulty in falling asleep, waking up too early, frequent awakening with difficulty in falling asleep again and secondary daytime impairment related to nighttime sleep difficulties (23).

The aim of this study was to assess the prevalence of insomnia in liver cirrhosis patients without overt hepatic encephalopathy using the ICSD-2 definition (23) to assess the association between the insomnia and the liver cirrhosis severity and to assess the association between insomnia and the underlying etiology of liver cirrhosis.

## Methods

This was a cross-sectional study conducted at King Abdulaziz Medical City (KAMC), Riyadh, over a period of six months between January 2012 and July 2012. The Institutional Review Board (IRB) at King Abdullah International Medical Research Center (KAIMRC), Riyadh, approved this study.

Data collection was carried out by personal professional interviews (EA) using a structured questionnaire. These questionnaires were adopted from validated international questionnaires and were used previously in patients with renal failure (24). We enrolled all stable patients with confirmed diagnosis of liver cirrhosis who were being followed at the hepatology and pre-liver transplant clinics. We excluded patients with other comorbidity that may cause sleep disturbance, including chronic pulmonary diseases and congestive heart failure.

The primary physicians identified all patients with confirmed diagnosis of liver cirrhosis and classified them according to the severity of liver cirrhosis based on the CTP scores (25). The diagnosis of liver cirrhosis was based on liver radiological studies, liver biopsy when available and compatible clinical data as per the diagnosis of the hepatologist who referred the case for study. The patients who agreed to participate were introduced by the primary physician to the study co-investigator who interviewed the patients, obtained the consents and reviewed all the questionnaires with the participants.

Insomnia was assessed using the ICSD-2 definition (23). Moreover, patients were also screened for depression symptoms using a rapid screening questionnaire developed for medical patients (26,27).

In addition we gathered demographic data and information pertinent to liver cirrhosis, such as the underlying cause of liver cirrhosis and the severity of liver cirrhosis based on the CTP score (25,27).

## Statistical analysis

The collected data were transferred and analyzed using SAS version 9.2 (SAS Institute Inc., Cary, NC). The mean and standard deviation were used to summarize age, neck size and BMI. Counts and percentages were used to summarize the demographic and clinical characteristics such as gender, occupation, smoking status, depression and insomnia. Chi-squared test/t-test was used to test the associations/differences between the demographic/clinical characteristics and the presence of insomnia (Table 1). Also insomnia and its association with sleep patterns were examined by Chi-squared test (Table 1). Stepwise logistic regression was used to determine the factors associated with the presence of insomnia (Table 2). P-values less than 0.05 were considered significant.

**Table 1:** Insomnia and its association with demographic/clinical characteristics.

Characteristics	Levels		Insomnia 84(42%)		No insomnia 116(58%)		

		n(%)	n	%	n	%	P-value

Gender	Female	85(42.5)	42	49.4	43	50.6	0.068
	Male	115(57.5)	42	36.5	73	63.5	

Education	Illiterate	69(34.5)	24	34.8	45	65.2	0.133
	Non-illiterate	131(65.5)	60	45.8	71	54.2	

Occupation	Employed	22(11.0)	7	31.8	15	68.2	0.305
	Unemployed	178(89.0)	77	43.3	101	56.7	

Smoking	Yes	159(79.5)	69	43.4	90	56.6	0.431
	No	41(20.5)	15	36.6	26	63.4	

Coffee Intake	Yes	58(29.0)	33	56.9	25	43.1	0.006^*^
	No	142(71.0)	51	35.9	91	64.1	

Depression	Yes	59(29.5)	12	20.3	47	79.7	0.001^*^
	No	141(70.5)	72	51.1	69	48.9	

HTN	Yes	42(21.0)	19	45.2	23	54.8	0.632
	No	158(79.0)	65	41.1	93	58.9	

Cause of liver cirrhosis	B	38(19.4)	14	36.8	24	63.2	0.001^*^
	C	118(60.2)	61	51.7	57	48.3	
	Others	40(20.4)	6	15.0	34	85.0	

CTP	A	80(40.0)	44	55.0	36	45.0	0.009^*^
	B	84(42.0)	27	32.1	57	67.9	
	C	36(18.0)	13	36.1	23	63.9	

EDS	*ESS* >*10, Yes*	59(29.5)	39	66.1	20	33.9	0.001^*^
	*ESS* ≤ *10, No*	141(70.5)	45	31.9	96	68.1	

Cannot sleep within 30 minutes	*Yes*	141(70.5)	72	51.1	69	48.9	0.001^*^
	*No*	59(29.5)	12	20.3	47	79.7	

Sleep duration	< *5 hours*	86(43.0)	48	55.8	38	44.2	0.002^*^
	*6–7 hours*	102(51.0)	31	30.4	71	69.6	
	≥*8 hours*	12(6.0)	5	41.7	7	58.3	

Age	*Mean*±*SD*	58.9±12.2	61.6±12.0		57.0±12.0		0.008#
BMI	*Mean*±*SD*	27.7±5.7	27.7±5.7		27.7±5.8		0.970
Neck size	*Mean*±*SD*	37.2±4.0	37.8±4.2		36.7±3.7		0.043#

# The t-test statistic is significant at the .05 level. ^*^The Chi-square statistic is significant at the .05 level.

**Table 2:** Multivariate regression identified the factors associated with the presence of insomnia.

Parameter	Levels	Estimate	SE	P-value	OR	95% Confidence Limits	

Intercept		-1.08	0.40	0.007			
Coffee Intake	Yes	0.50	0.21	0.020^*^	2.7	1.167	6.252
Depression	Yes	-0.63	0.26	0.014^*^	0.3	0.103	0.778
Cause of liver cirrhosis	B	0.34	0.36	0.352	4.4	1.108	17.711
Cause of liver cirrhosis	C	0.82	0.30	0.006^*^	7.2	2.169	23.776
CTP	A	0.60	0.29	0.034^*^	1.9	0.621	5.609
CTP	B	-0.58	0.28	0.036^*^	0.6	0.193	1.675
EDS	Yes	0.84	0.22	0.001^*^	5.3	2.239	12.706
Cannot sleep within 30 minutes	Yes	0.79	0.24	0.001^*^	4.9	1.923	12.33
Sleep duration	≤ 5 hours	1.06	0.38	0.006^*^	5.7	2.398	13.386
Sleep duration	≥*8 hours*	-0.38	0.60	0.533	1.4	0.223	8.217

^*^The Wald Chi-square statistic is significant at the .05 level.

## Results

The total participants with liver cirrhosis enrolled in this study were 200 patients. The mean age was 58.9 (SD ± 12.2) years (range 20–88 years), and 115 patients were men (57.5%). Mean BMI was 27.7 (SD ± 5.7) kg/m^2^ and mean neck size was 37.2 (SD ± 4.0) cm. The majority (79.5%) of patients were smoking cigarettes, and (29%) were depressed. Table 1 shows other demographic characteristics of the patients. The cause of liver cirrhosis was hepatitis C in the majority of the cases (60.2%), hepatitis B in 19.4%, and 20.4% due to other causes. Based on CTP liver severity score, 40% of the patients were CTP class A, 42% were class B and 18% were class C. In our research, the prevalence of insomnia among patients with liver cirrhosis was 42%.

**Figure 1:**
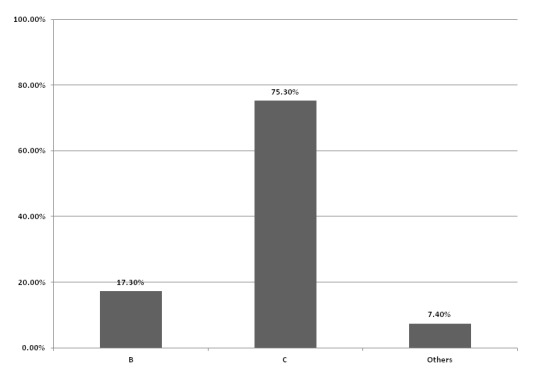
The percents of insomnia among patients with liver cirrhosis across cause of liver cirrhosis.

**Figure 2:**
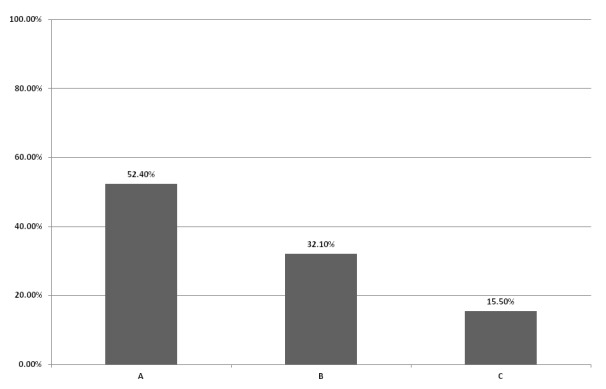
The percents of insomnia among patients with liver cirrhosis across CTP.

We compared the characteristics and risk factors of patients with insomnia to patient with no insomnia among liver cirrhosis patients (Table 1). Liver cirrhosis patients with insomnia were significantly older (61.6 ± 12.0 years) than non-insomniac patients (59 ± 12.0 years), p-value = 0.008. However, insomnia was not associated with gender (p = 0.068) and smoking habits (p = 0.431). Depression was less common among insomnia patients (20.3% vs. 51.1%, p = 0.001) while coffee intake was significantly more among liver cirrhosis with insomnia compared to those with no insomnia (56.9% vs. 35.9%, p = 0.006). Insomnia, was common among hepatitis-C patients compared to hepatitis-B and other hepatitis (51.7% vs. 36.8% and 15%, p-value = 0.001). Figure 1 shows that insomnia was more common among patients with hepatitis C compared to hepatitis B, and other hepatitis. Insomnia was more common among CTP-A (55.0%) compared to CTP-B (32.1%) and CTP-C (36.1%), p = 0.009 Figure 2. Patients who had excessive daytime sleepiness (EDS) experienced insomnia more frequently than those who didn’t (66.1% vs. 31.9%, p-value = 0.001). Patients who sleep within 30 minutes experienced insomnia less frequently than those who didn’t (20.3% vs. 51.1%, p-value = 0.001). Patients who slept five hours or less (55.8%) or 8 hours or more (41.7%) a night were more likely to suffer from insomnia compared to patients who slept 6–7 hours (30.4%), p-value = 0.002. As shown in (Table 1), we compared the presence of insomnia with the absence of insomnia by age, BMI, and neck size. Insomniac patients were significantly older than non-insomniac (61.6 ± 12.0 vs. 57.0 ± 12.0 years, p = 0.008). Neck size in patients with insomnia was larger than patients without insomnia (37.8 ± 4.2 vs. 36.7 ± 3.7, p-value = 0.043). No difference in BMI was apparent between the insomniac and non-insomniac groups (p-value = 0.970).

Multivariate risk factors and presence of insomnia in patients with liver cirrhosis were analyzed by stepwise logistic regression, see Table 2. There was significant association between presence of insomnia and hepatitis C as compared to other hepatitis (adjusted OR = 7.2; p-value = 0.006). Severity of liver cirrhosis CTP-A had a significantly higher prevalence of insomnia as compared to CTP-C (adjusted OR = 1.9; p-value = 0.034). There was significant association between presence of insomnia and excessive daytime sleepiness (adjusted OR = 5.3; p-value = 0.001). Patients who slept 5 hours or less a night were 5.7 times more likely to suffer from insomnia compared to patients who slept 6–7 hours (adjusted OR = 5.7; p-value = 0.006).

## Discussion

Sleep disturbances reported in liver cirrhosis in previous studies were mainly about circadian rhythm disturbances. Different methods are used in reporting sleep disturbances among liver cirrhosis patients. This makes it difficult to compare different studies relative to the prevalence of sleep disturbance, particularly insomnia and other circadian sleep abnormalities. To the best of our knowledge, no similar studies exist that investigate the prevalence of insomnia in patients with liver cirrhosis using ICSD-2 definition. (23) This study is the largest study that compared sleep disturbance, particularly insomnia and sleep patterns among liver cirrhosis patients. The prevalence of insomnia in this study was high. Cordoba and colleagues reported higher sleep disturbance among liver cirrhosis 47.7% compared to chronic renal failure patients 38.6% and healthy control 4.5% (1). In this study the prevalence of insomnia among liver cirrhosis without evidence of hepatic encephalopathy was high: 42%, and it was lower than the prevalence of insomnia in dialysis patients that we reported previously 60.8% (24). Compared to a study by Mostacci and colleagues (2), we did find an inverse relationship to the severity of liver cirrhosis. Patients with CPS-A had more insomnia compared to CPS-C. In this study insomnia was common among hepatitis-C patients compared to hepatitis B. This has also been observed in other studies, which documented higher prevalence of sleep disturbances among liver cirrhosis patient secondary to hepatitis C (5,21,28). It was suggested that the changes in immunologic function leading to increase circulating cytokines in patients with hepatitis C may be responsible for sleep disturbances (5).

Compared with the study by Mostacci and colleagues (2), which showed that EDS as assessed by ESS was not different between healthy and patients, in our in our study EDS was prevalent among insomnia patients. Similar to other studies, we did not find a correlation between the severity of liver cirrhosis as assessed by CPS and insomnia among liver cirrhosis cases (1,2,29). Depression was less common among those with insomnia than normal which indicates that depression per se is not contributing to insomnia in cirrhosis patients. Short-sleep duration was associated with insomnia in this study, which probably confirms consistency of our findings.

One limitation of our study is that it was not controlled. Another limitation was that we assessed insomnia subjectively: we did not use sleep diaries or wrist actigraphy to objectively assess insomnia.

## Conclusion

In conclusion, there is a significant association between liver cirrhosis patients without overt hepatic encephalopathy and sleep disturbances. Insomnia, delayed-phase sleep and excessive daytime sleepiness were common among liver cirrhosis patients. Greater attention needs to be given to the care of liver cirrhosis patients with regard to the diagnosis and management of insomnia and other associated sleep disorders.
